# Clinical Specimens are the Pool of Multidrug- resistant Pseudomonas aeruginosa Harbouring oprL and toxA Virulence Genes: Findings from a Tertiary Hospital of Nepal

**DOI:** 10.1155/2021/4120697

**Published:** 2021-10-29

**Authors:** Yamuna Chand, Sujan Khadka, Sanjeep Sapkota, Suprina Sharma, Santosh Khanal, Alina Thapa, Binod Rayamajhee, Dhruba Kumar Khadka, Om Prakash Panta, Dipendra Shrestha, Pramod Poudel

**Affiliations:** ^1^Department of Microbiology, National College, Tribhuvan University, Kirtipur, Nepal; ^2^State Key Laboratory of Environmental Aquatic Chemistry, Research Center for Eco-Environmental Sciences, Chinese Academy of Sciences, Beijing 100085, China; ^3^University of Chinese Academy of Sciences, Beijing 100049, China; ^4^State Key Laboratory of Respiratory Disease, Guangzhou Institutes of Biomedicine and Health, Chinese Academy of Sciences, Guangzhou 510530, China; ^5^Central Department of Microbiology, Tribhuvan University, Kirtipur, Nepal; ^6^State Key Laboratory of Alpine Ecology and Biodiversity, Institute of Tibetan Plateau Research, Chinese Academy of Sciences, Beijing 100101, China; ^7^School of Optometry and Vision Science, Faculty of Medicine and Health Sciences, UNSW, Sydney, Australia; ^8^Department of Microbiology, Bir Hospital, National Academy of Medical Sciences (NAMS), Kathmandu, Nepal; ^9^Central Department of Biotechnology, Tribhuvan University, Kirtipur, Nepal; ^10^University Grants Commission, Bhaktapur, Nepal

## Abstract

The multidrug- or extensively drug-resistant (MDR/XDR) *Pseudomonas aeruginosa* carrying some virulence genes has become a global public health threat. However, in Nepal, there is no existing report showing the prevalence of *oprL* and *toxA* virulence genes among the clinical isolates of *P*. *aeruginosa*. Therefore, this study was conducted for the first time in the country to detect the virulence genes (*oprL* and *toxA*) and antibiotic susceptibility pattern of *P*. *aeruginosa*. A total of 7,898 clinical specimens were investigated following the standard microbiological procedures. The antibiotic susceptibility testing was examined by the modified disc diffusion method, and virulence genes *oprL* and *toxA* of *P*. *aeruginosa* were assessed using multiplex PCR. Among the analyzed specimens, 87 isolates were identified to be *P*. *aeruginosa* of which 38 (43.68%) isolates were reported as MDR. A higher ratio of *P*. *aeruginosa* was detected from urine samples 40 (45.98%), outpatients' specimens 63 (72.4%), and in patients of the age group of 60–79 years 36 (41.37%). *P*. *aeruginosa* was more prevalent in males 56 (64.36%) than in female patients 31 (35.63%). Polymyxin (83.90%) was the most effective antibiotic. *P. aeruginosa* (100%) isolates harboured the *oprL* gene, while 95.4% of isolates were positive for the *toxA* gene. Identification of virulence genes such as *oprL* and *toxA* carrying isolates along with the multidrug resistance warrants the need for strategic interventions to prevent the emergence and spread of antimicrobial resistance (AMR). The findings could assist in increasing awareness about antibiotic resistance and suggest the judicious prescription of antibiotics to treat the patients in clinical settings of Nepal.

## 1. Introduction

*Pseudomonas aeruginosa* is known as one of the most widely spread opportunistic human pathogens causing 18 to 63% of infections worldwide [1, 2]. It can grow at a temperature of 42°C, this unique character helps to differentiate it from the rest of the *Pseudomonas* species [3]. Most strains produce water-soluble pigments, such as pyocyanin, pyoverdin, pyorubin, and pyomelanin [4, 5]. The ability of *P*. *aeruginosa* to grow in minimum nutritional requirements and to withstand various physical conditions such as disinfectants has assigned this organism to persist both in hospital and community settings [6–8]. *P*. *aeruginosa* commonly caused a high rate of infection in immunocompromised and cystic fibrosis (CF) patients [9].

*P. aeruginosa* has great diversity and is capable of causing life-threatening contagious infections in a multifariousness patients' population [10], causing several diseases such as urinary tract infections (UTIs) [11], respiratory tract infections (RTIs) [12], burn wounds, skin and soft tissue infections [13], bacterial keratitis [14], and swimmer ear infections [15]. The pathogenic effect of *P. aeruginosa* is mainly mediated by secreted virulence factors [10]. There are several extracellular and cell-associated virulence factors that may lead to its pathogenicity. The colonization of these factors can cause bloodstream invasive, extensive tissue damage, and dissemination [10]. The pathogenesis of *P*. *aeruginosa* is also linked with the extracellular and cell-mediated virulence factors such as *toxA, exoS, exoY, exoU, oprL, oprI, lasA, lasB, oprD, plcH, plcN*, and *nan1* which can cause host tissue destruction, invasion, and spread in the host body [16]. Virulence genes such as *oprL, oprI*, and *oprD* are the major constituents of outer membrane lipoproteins of *P*. *aeruginosa* which are also used as markers for the identification of *P*. *aeruginosa*-associated infections [17]. Similarly, the *toxA* gene is one of the virulence genes, which encodes exotoxin A produced by *P*. *aeruginosa* and inhibits protein biosynthesis by stopping the elongation of polypeptide chains [17–19].

Increasing antibiotic resistance is a global problematic issue in recent days, and drug resistance in *P. aeruginosa* has become a huge concern due to its ability to cause frequent nosocomial as well as chronic infections [6]. Resistance to the different classes of antibiotics by *P. aeruginosa* is due to the loss or reduced copy numbers of *oprD* and overproduction of active efflux pumps, AmpC *β*-lactamase and extended-spectrum *β*-lactamases and their resistance mechanism are classified as intrinsic, acquired, and adaptive [6]. In Nepal, the increasing trend of antimicrobial-resistance bacteria in both clincal and nonclinical settings [11, 12, 20–26] is becoming an alarming health issue. There is no exact observable scheme for marking antibiotic resistance patterns and their use in Nepal. Additionally, few studies and some available secondary data are not sufficient to study the current scenario and it is hard to describe the true positive trends of antibiotic resistance of *P. aeruginosa* in Nepal. Therefore, this cross-sectional study is designed to assist the current resistant pattern of *P. aeruginosa* against different classes of antibiotics and to identify the involvement of several virulence genes in resistant mechanisms by using polymerase chain reaction (PCR) which ultimately helps to select appropriate antibiotics useful for the treatment of infectious disease caused by *P. aeruginosa*.

## 2. Materials and Methods

### 2.1. Study Period, Design, and Sample Size

This cross-sectional study was conducted between November 2018 and April 2019 at a public hospital in Nepal. In total, 7898 different clinical specimens comprising urine (*n* = 4555), sputum (*n* = 975), pus (*n* = 595), blood (*n* = 1241), and body fluids (*n* = 532) were collected. Sample collection was carried in the sterile, leak-proof, clearly labeled container and processed immediately. Only sufficiently collected and properly labeled samples were included in this study to avoid the error in results. Clinical samples with missing demographic information such as name, age, and gender were excluded from this study.

### 2.2. Sample Processing and Identification of Isolates

All collected clinical specimens were cultured semiquantitively on several culture media such as nutrient agar, nutrient broth, blood agar, MacConkey agar, cysteine-lactose electrolyte deficient (CLED) medium, brain heart infusion (BHI) medium, and chocolate agar (CA). The bacterial count more than >10^5^ CFU/mL is referred to as significant bacteriuria [27]. The cultural media were bought from HiMedia Laboratories, India. Further confirmation of *Pseudomonas* sp. was carried out by using cetrimide agar medium and following the standard microbiological procedures that included colonial morphology, Gram staining, and a series of standard biochemical tests (indole, methyl red, Voges–Proskauer, citrate utilization, urease, oxidative/fermentative, catalase, oxidase, triple sugar iron, mannitol fermentation, and motility) [28, 29].

### 2.3. Antibiotic Susceptibility Testing

The antibiotic susceptibility testing was performed on Muller Hinton agar (MHA) by the modified disc diffusion method according to the criteria set by the Clinical and Laboratory Standards Institute (CLSI), 2018 [30, 31]. The antibiotics disks used in this study were procured from HiMedia Laboratories, India, and include ceftazidime (30 *µ*g), ciprofloxacin (5 *µ*g), imipenem (10 *µ*g), tobramycin (10 *µ*g), piperacillin (100 *µ*g), piperacillin-tazobactam (100/10 *µ*g), nitrofurantoin (300 *µ*g), gentamicin (10 *µ*g), norfloxacin (10 *µ*g), aztreonam (30 *µ*g), cefixime (5 *µ*g), carbenicillin (100 *µ*g), and polymyxin (300 *µ*g). Strains were considered multidrug resistant (MDR) if they were resistant to at least one agent in three or more antimicrobial categories [32].

Following CLSI guidelines, the quality of MHA plate and antibiotic discs was checked from their lot number, manufacturer, expiry date, and proper storage condition. The aseptic condition was maintained during the collection and processing of the specimens to avoid any contamination from an outlying area. Likewise, the quality of each batch of culture and biochemical media was assured by incubating a randomly sampled medium at 37°C for 24 h. A reference strain *Pseudomonas aeruginosa* ATCC 27853 was used to maintain the quality control of AST. In the same way, the thickness and pH of MHA were kept at 4 mm and 7.2–7.4, respectively.

### 2.4. DNA Extraction and Detection of Virulence Genes Using PCR

For the identification of virulence genes (*oprL* and *toxA*), bacterial DNA was extracted from each *P*. *aeruginosa* isolate by phenol-chloroform assay [33]. The PCR amplification was carried out by using a temperature gradient thermal cycler (PCR tube 96 wells, Takara/Japan) with a specific forward and reverse primer for the detection of *oprL* and *toxA* genes, respectively. The primer was then diluted to a working concentration of 10 Pm by using nuclease-free water (Table 1).

The PCR was carried out in total 20 *µ*L volume of reaction mixture containing 2 *µ*L of template DNA, 1 *µ*L of each primer, 4 *µ*L of the master mixture, and 12 *µ*L of nuclease-free water and Taq-polymerase enzyme with 35 cycles. The annealing temperature was 61.8°C for *oprL* and 58.2°C for *toxA*. The PCR condition is depicted in Table 1. The PCR products were separated by gel electrophoresis on 1% agarose gel containing 5 *µ*L of ethidium bromide. The band of size about 504 bp and 352 bp of *oprL* and *toxA* was produced, respectively, along with 100 bp DNA marker and positive control [18, 34]. *Pseudomonas aeruginosa* ATCC 27853 harbouring both *toxA* and *oprL* genes was considered as the positive control, while the negative control was maintained by using nuclease-free water.

### 2.5. Statistical Analysis

Data analysis was performed using the R-programming statistical analysis tool (version 1.2.5033) and Statistical Package for Social Sciences (SPSS) software (Version 16.0). The chi-square test (*χ*^2^) was estimated between the clinical, sociodemographics of patients with the distribution of MDR *P. aeruginosa*, and the statistically significant associations were represented by *P* < 0.05^*∗*^ (significant), while *P* > 0.05 refers insignificance. The graphical presentation was performed using the ggplot package (grammar of graphics) (version 3.3.2) of R-programming language.

## 3. Results

### 3.1. Growth Pattern

**Figure 1:**
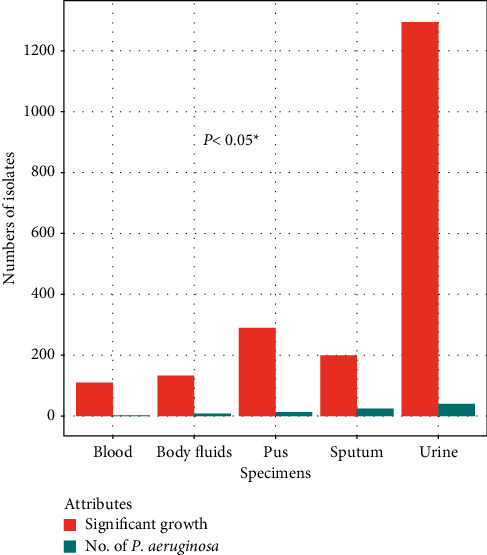
Distribution of *P*. *aeruginosa* among different clinical specimens. The isolation of *P. aeruginosa* and their significant growth from different specimens are significantly associated (*P* < 0.05^*∗*^).

Of the 7898 specimens examined, 2026 (26%) showed significant growth (i.e., >10^5^ CFU/mL); among them, 87 (4.29%) were positive for *P*. *aeruginosa*. The highest significant growth (1294) was observed in urine samples, while the lowest significant growth (110) was noted from the blood samples. Likewise, the highest numbers of *P. aeruginosa* (40) were also recovered from the urine samples, whereas the lowest numbers of them (2) were isolated from the blood samples (Figure 1).

### 3.2. Clinical and Sociodemographic Characteristics of Patients and Distribution of MDR *P. aeruginosa*

The highest number of *P*. *aeruginosa* was isolated from 56 (64.36%) male patients, and community-acquired infections were found to be greater which were detected from 63 (72.41%) patients. A substantial number (36 (41.37%)) of *P*. *aeruginosa* were isolated from the age group 60–75. The highest percentage of *P*. *aeruginosa* was isolated from urine samples 40 (45.98%) followed by sputum 24 (27.59%). The occurrence of MDR *P*. *aeruginosa* was higher in females, inpatients, and 40–59 years age group patients (*P* > 0.05). In addition, the maximum number of MDR *P*. *aeruginosa* (MDRPA) was isolated from urine 24 (60.00%) followed by sputum 8 (33.33%), pus 4 (30.77%), and body fluids 2 (25.00%) (*P* > 0.05) (Table 2).

### 3.3. Susceptibility to Antimicrobial Agents

The antibiotic susceptibility pattern of *P. aeruginosa* isolates revealed that polymyxin, tobramycin, gentamicin, imipenem, and ceftazidime were the most effective antibiotics *in vitro* with sensitivities of 73 (83%), 71 (81.60%), 69 (79.31%), 63 (72.41%), and 61 (70.11%), respectively. On the other hand, all the *P. aeruginosa* isolates were resistant (100%) to cefixime. The antibiotic susceptibility patterns of *P. aeruginosa* isolates are depicted in Table 3. Out of 87 *P*. *aeruginosa* isolates, 38 (43.68%) were identified to be MDR.

### 3.4. Virulence Genes Associated with *P. aeruginosa*

**Figure 2:**
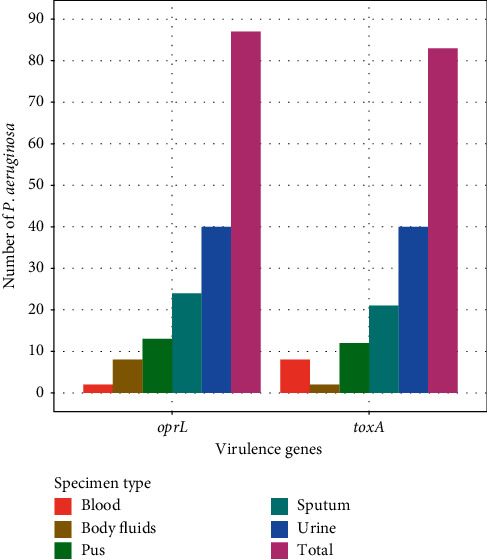
Prevalence of virulence genes (*oprL* and *toxA*) in *P. aeruginosa*.

**Figure 3:**
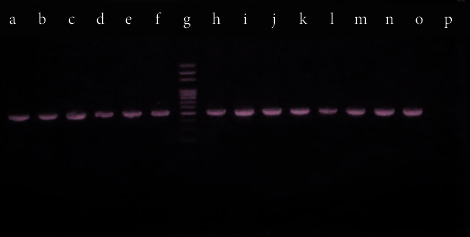
Agarose gel (1%) electrophoresis of PCR assay of the *oprL* gene (504 bp). a b, c d, e f, h i, j k, l m, n, and o: *oprL*-positive *P. aeruginosa* isolates, g: 100 bp marker, o: positive control, and p: negative control (nuclease-free water).

**Figure 4:**
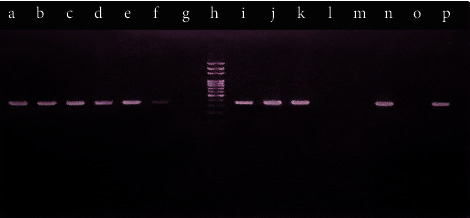
Agarose gel (1%) electrophoresis of PCR assay of the *toxA* gene (352 bp). a b, c d, e f, i j, k, and n: *toxA*-positive *P. aeruginosa* isolates, g, l, and m: *toxA*-negative *P. aeruginosa* isolates, h: 100 bp marker, o: negative control (nuclease-free water), and p: positive control.

Out of 87 total isolates, 83 (95.4%) *P. aeruginosa* isolates showed the presence of *toxA* virulence genes whereas the *oprL* gene was detected in all the collected *P*. *aeruginosa* isolates, 87 (100%) (Figure 2). The detection of virulence genes of *P*. *aeruginosa* was performed by multiplex PCR. The present study showed that, of 87 tested *P*. *aeruginosa* isolates, 87 (100%) contained the *oprL* gene (sensitivity = 100%) whereas other species of bacteria did not produce any positive result (specificity = 100%), while the amplification of the *toxA* gene showed that, of 87 tested *P*. *aeruginosa* isolates, 83 (95.40%) contained the *toxA* gene (sensitivity = 95%) whereas other species of bacteria did not yield any positive result (specificity = 100%). PCR amplification of the *toxA* and *oprL* genes is shown in Figures 3 and 4.

## 4. Discussion

*P*. *aeruginosa* is associated as a versatile opportunistic human pathogen, and its ultimate infection is reported to be accomplished by attachment, colonization, local invasion, and dissemination as a systemic disease [35, 36]. In this study, the prevalence of *P*. *aeruginosa* isolates was 4.29% which is in line with the study conducted in Nepal where the prevalence rate was 5.10% [37]; however, the prevalence rate was lower (2.75%) in the study performed in Pakistan [38]. This might be due to the types of studied populations, different geographical locations, and types of hospitals. The distribution of *P*. *aeruginosa* was higher in male patients, 56 (64.36%), than in female patients, 31 (35.63%). The possible reasons may be males have routine outdoor work and they are frequently at risk of infection from the infected environments [39]. The prevalence rate of infections was higher in outpatients, 63 (72.41%), compared to the hospital-admitted patients, 24 (27.58%), which may be due to frequent exposure of the outpatients to the infected environment. The occurrence of *P*. *aeruginosa* isolates was greater in the age group of 60–79 years with a prevalence rate of 41.37% which illustrates that the infection caused by *P*. *aeruginosa* is more common in patients of the old-age group. This could be associated with the decrease in the function of the immune system and prolonged duration of hospitalization [37, 40, 41]. Moreover, the occurrence of MDR *P*. *aeruginosa* was higher in females, inpatients, and 40–59 years age group patients (*P* > 0.05). The higher percentage of MDR *P*. *aeruginosa* was isolated from urine samples (*P* > 0.05). The lack of national antibiotic management policies and easy access to antibiotics without the prescription of the medical personnel might have contributed to the emergence of MDR isolates [42].

The most effective drug for *P*. *aeruginosa* isolates was found to be polymyxin 73 (83.90%), also called the last resort antibiotic for the Pseudomonadaceae family in the hospitals, and the less effective antibiotic was cefixime 87 (100%). Out of 87 *P*. *aeruginosa* isolates, 38 (43.68%) were identified to be MDR. This was in line with the study conducted in India where the prevalence of the MDRPA was 50.00% [43]. The development of antibiotic resistance towards *P*. *aeruginosa* might be due to random use of antibiotics, production of different types of enzyme-like carbapenemases, AmpC-lactamases, quorum sensing modification of different target sides, etc. [17, 44]. Furthermore, one of the major causes of the emergence of *P*. *aeruginosa* is prescribing antibiotics without performing susceptibility tests due to the lack of laboratory facilities in most of the healthcare centers in Nepal [37, 39, 45, 46].

The PCR results showed that 87 (100%) of 87 *P*. *aeruginosa* isolates were positive for *oprL* genes. Similarly, in this study, 83 (95.40%) of 87 *P*. *aeruginosa* were positive for the *toxA* gene. Almost a comparable study was carried out in Brazil reporting 81.82% *P*. *aeruginosa* isolates were positive for *toxA* and 100% for *oprL* genes [47]. Similarly, a study conducted in Iran reported 69.4% of *P*. *aeruginosa* isolates were positive for the *toxA* gene [48]. The divergences in the distribution of virulence factor genes in the different populations might be due to the probability that some *P*. *aeruginosa* strains are better adapted to the conditions found in infectious sites that may be returned to the diverse geographical and environmental sources. The prevalence of *P*. *aeruginosa* and its virulence genes depends on various causes consisting the nature of places, degree of contamination and type, immune status of individual patients, and virulence of strains [49].

Exotoxins A are either actively secreted through the type 1 secretion system (T1SS), the type 2 secretion system (T2SS), and the type 3 secretion system (T3SS) or passively secreted via the cell [45]. The exotoxin A is encoded by a gene called *exoA* which is involved in tissue necrosis and resistant to antibiotics [46]. The L and I are two outer membrane lipoproteins of *P*. *aeruginosa* found only in this organism, so they could be a suitable factor for the rapid identification of *P*. *aeruginosa* in clinical specimens. This bacterium is also answerable for inherent resistance to antiseptics and antibiotics [49]. Moreover, the detection of virulence genes of *P*. *aeruginosa* using multiplex PCR showed high sensitivity and specificity which revealed that multiplex PCR may be one of the rapid diagnostic tools for the identification of *P*. *aeruginosa* infections.

## 5. Conclusions

In this research, polymyxin and aminoglycosides were found to be effective antibiotics for treatment, and the studies revealed that almost all *P*. *aeruginosa* harbor both *oprL* and *toxA* genes. The higher prevalence of MDR *P*. *aeruginosa* in clinical specimens is worrisome, and special attention is required in regular surveillance of antibiotic susceptibility patterns along with their careful and judicious use. Likewise, the presence of intrinsic virulence and pathogenicity of *P*. *aeruginosa* is indicated by the existence of virulence genes such as *oprL* and *toxA*, so detection of these genes by PCR is highly recommended.

## Figures and Tables

**Table 1:** Nucleotide sequence of primers and condition used to amplify species-specific virulence genes in *P*. *aeruginosa* by PCR.

Virulence factors	Target genes	Primer names	Sequence (5′ to 3′)	Annealing temperature (°C)	Amplicon size (bp)	Refs
**Exotoxin A**	*toxA*	*toxA*-f	GGTAACCAGCTCAGCCACAT	58.2	352	[34]
		*toxA*-r	TGATGTCCAGGTCATGCTTC			

**Sialidase enzyme**	*oprL*	*oprL*-f	ATGGAAATGCTGAAATTCGGC	61.8	504	[18]
		*oprL*-r	CTTCTTCAGCTCGACGCGACG			

**Table 2:** Clinical and sociodemographic characteristics of patients and distribution of MDR *P. aeruginosa*.

Attributes		*P. aeruginosa* isolates, *n* (%)	MDR, *n* (%)	*P* value
Gender	Male	56 (64.36)	23 (41.07)	0.512
	Female	31 (35.63)	15 (48.39)	

Age group (years)	1–19	6 (6.89)	3 (50.00)	0.23
	20–39	16 (18.39)	4 (25.00)	
	40–59	24 (27.58)	14 (58.33)	
	60–79	36 (41.37)	16 (44.44)	
	>79	5 (5.47)	1 (20.00)	

Status of patients	Inpatients	24 (27.58)	11 (45.83)	0.803
	Outpatients	63 (72.41)	27 (42.86)	

Sample types	Urine	40 (45.98)	24 (60.00)	0.063
	Sputum	24 (27.59)	8 (33.33)	
	Pus	13 (19.54)	4 (30.77)	
	Body fluids	8 (9.20)	2 (25.00)	
	Blood	2 (2.30)	0	

The significant association is represented by *P* < 0.05^*∗*^ (significant), while *P* > 0.05 refers insignificance.

**Table 3:** Antibiotic susceptibility pattern of *P*. *aeruginosa*.

Antibiotics	Antibiotic class	Mode of action	Sensitive, *n* (%)	Intermediate, *n* (%)	Resistant, *n* (%)
Aztreonam	Monobactams	Inhibition of cell wall synthesis	58 (66.66)	21 (24.13)	8 (9.19)
Ceftazidime	Cephalosporins		61 (70.11)	0 (0)	26 (29.88)
Cefixime			0 (0)	0 (0)	87 (100)
Carbenicillin	Penicillins		30 (34.48)	16 (18.39)	41 (47.12)
Piperacillin			54 (62.06)	10 (11.49)	23 (26.43)
Piperacillin-tazobactam	Penicillin combinations		37 (78.72)	1 (2.12)	9 (19.14)
Imipenem	Carbapenems		63 (72.41)	0 (0)	24 (27.58)
Polymyxin	Polypetides		73 (83.90)	14 (16.09)	0 (0)
Ciprofloxacin	Fluoroquinolones	Inhibition of nucleic acid synthesis	59 (67.81)	1 (1.14)	27 (31.03)
Norfloxacin			56 (64.36)	0 (0)	31 (35.36)
Gentamicin	Aminoglycosides	Inhibition of protein synthesis	69 (79.31)	0 (0)	18 (20.68)
Tobramycin			71 (81.60)	0 (0)	16 (18.39)
Nitrofurantoin	Nitrofurans	Inhibition of bacterial ribosomes and other macromolecules	0 (0)	0(0)	40 (100)

Note: The antimicrobial susceptibility of 40 isolates was tested against nitrofurantoin while the antimicrobial susceptibility of the remaining 47 isolates was performed against piperacillin-tazobactam.

## Data Availability

All data needed to support the results of this study are incorporated in the manuscript.

## Abbreviations

CLSI: Clinical and Laboratory Standards Institute
MDRPA: Multidrug-resistant *Pseudomonas aeruginosa*
MHA: Mueller Hinton agar.

## Additional Points

*Strengths and Limitations of the Study*. To the best of our knowledge, this is the first study determining the prevalence of *oprL* and *toxA* virulence genes harboring multidrug-resistant *P. aeruginosa* from clinical specimens in Nepal. Being unique of its kind, this study could assist the policymakers of the country to formulate the holistic approach for the proper implementation of the antimicrobial policies at all kinds of medical sectors, management of the treatment procedures, prescription of antibiotics, and improving the diagnostic procedures. These efforts might contribute to control the emergence and transmission of MDR bacteria in clinical settings. Furthermore, this research might be a significant reference for future studies on the prevalence of *P. aeruginosa* harboring *oprL* and *toxA* virulence genes in several other clinical settings of the country. Despite having numerous advantages, this study possesses some limitations such as the minimum inhibitory concentration test and *16S* rRNA sequencing for *P*. *aeruginosa* were not performed because of cost and unavailability of laboratories, respectively. Likewise, only *toxA* and *oprL* genes were taken as identification keys because of expensiveness. Additional studies might be required to confirm the pathogenicity and increasing trends of antibiotic resistance patterns of *P. aeruginosa*.
